# Supplementary material for: Identification and Functional Prediction of CircRNAs in Leaves of F1 Hybrid Poplars with Different Growth Potential and Their Parents
Source: Int J Mol Sci. 2023 Jan 23;24(3):2284. doi: 10.3390/ijms24032284 (PMC9916877; doi:10.3390/ijms24032284)
Supplement: Supplementary file 1 [file ijms-24-02284-s001.zip › Supplementary figures.pdf]

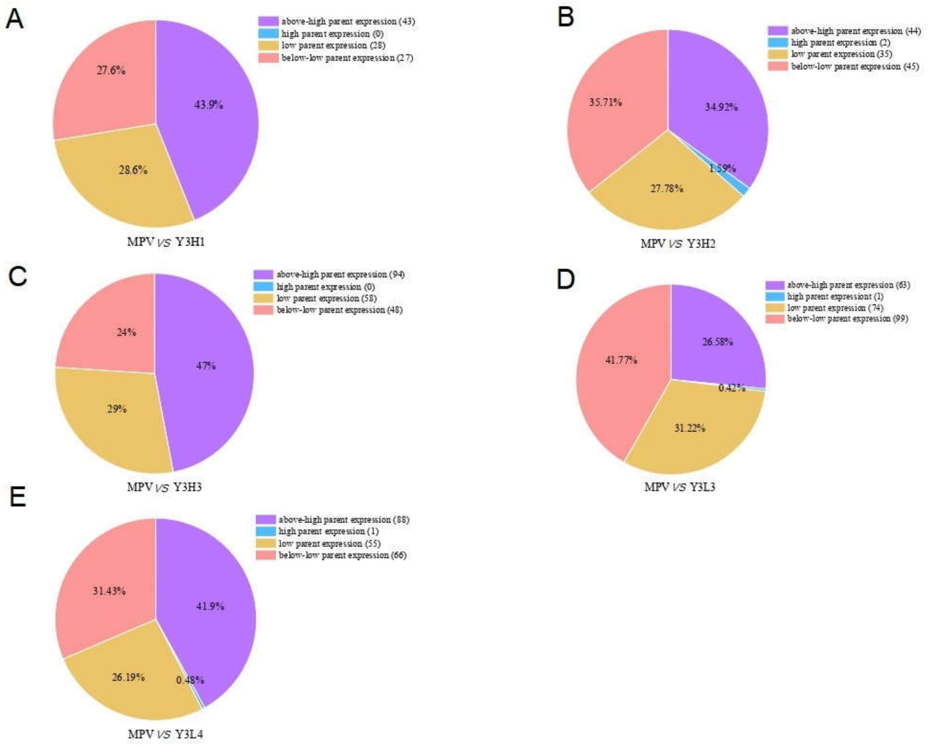


**Figure S1.** The number and proportion of above high parent expression, high parent expression, low parent expression and below low parent expression in non-additive expressed circRNAs of F1 hybrids with different growth potentials.

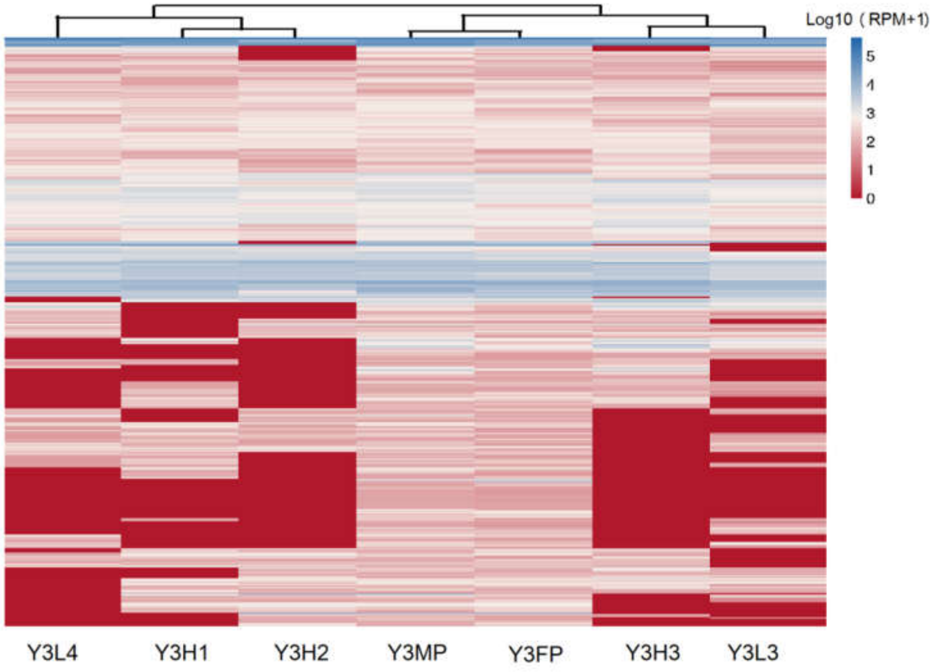


**Figure S2.** The expression level of co-expressed circRNAs between parents and F1 hybrids.

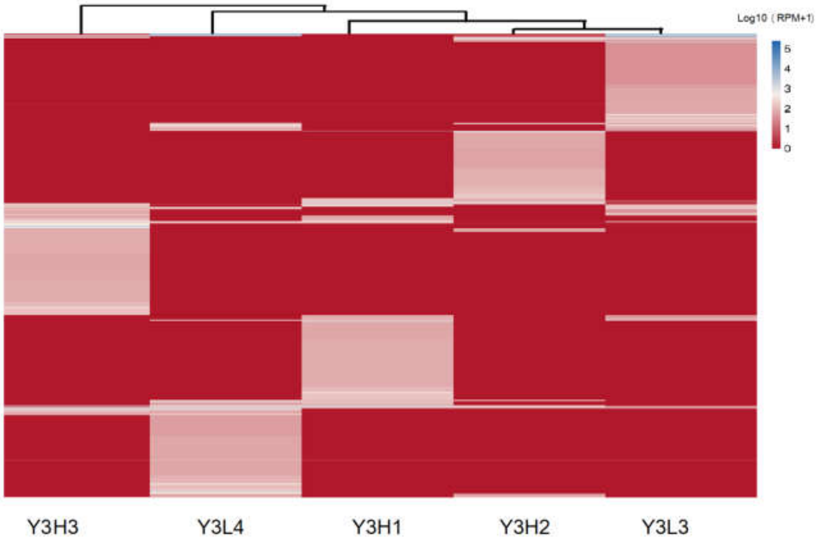


**Figure S3.** Expression level of specifically expressed circRNAs of F1 hybrids in F1 hybrids.

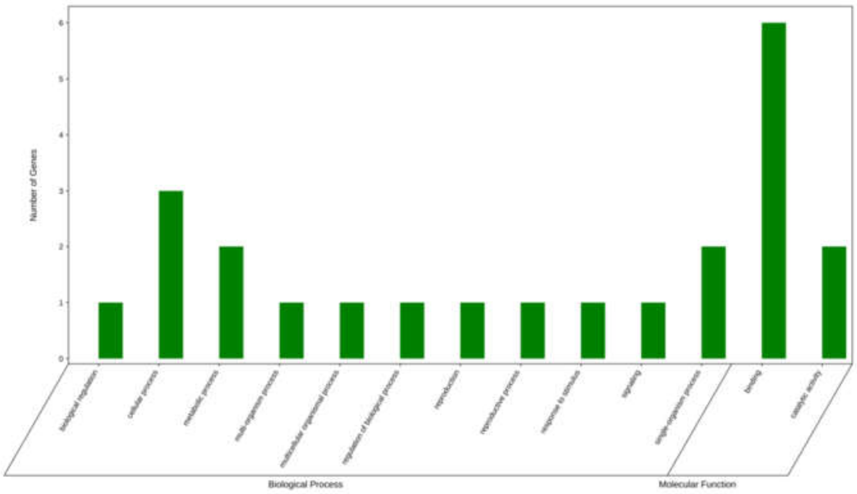


**Figure S4.** GO enrichment analysis of common DECs (4 control groups of F1 hybrids with high growth potential and F1 hybrids with low growth potential).

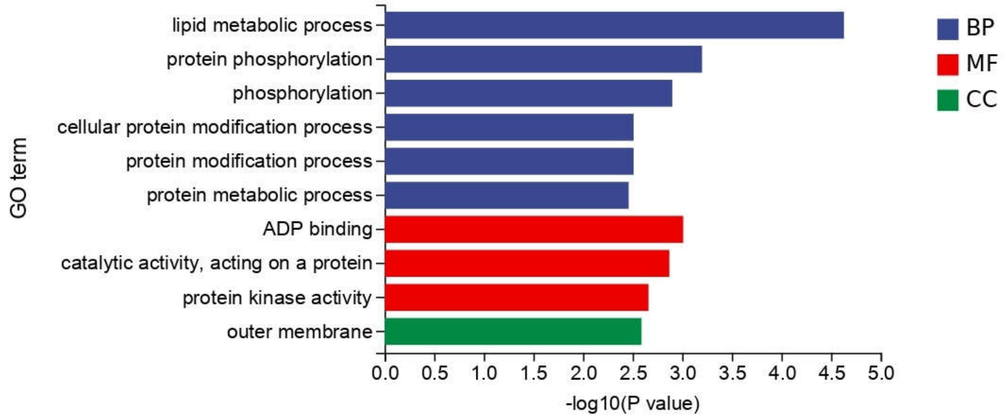


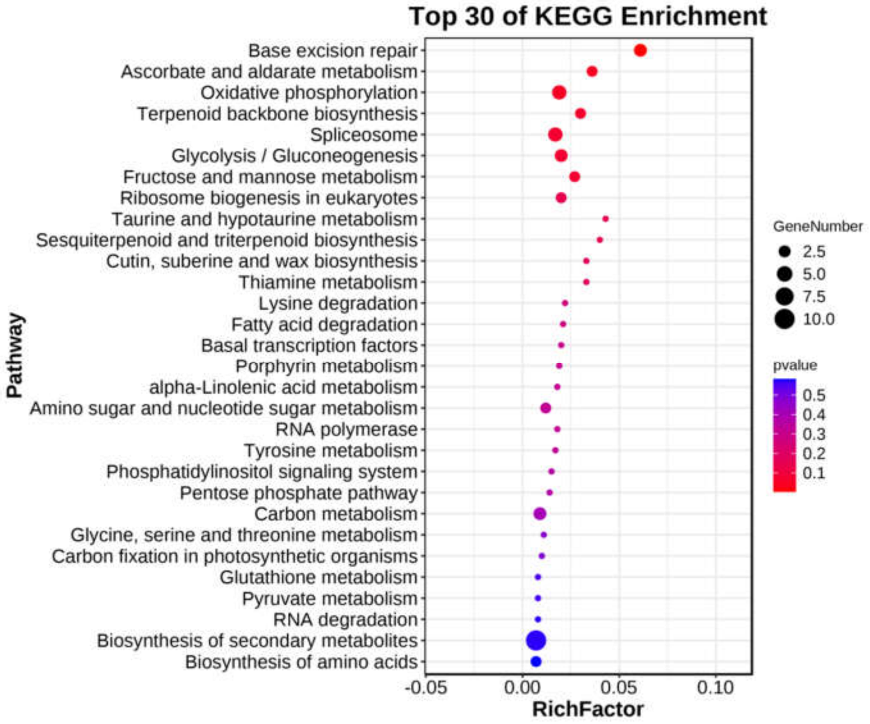


**Figure S5.** GO and KEGG enrichment analysis of protein coding genes of pink module. MF is molecular function, BP is biological process, and CC is cellular component.

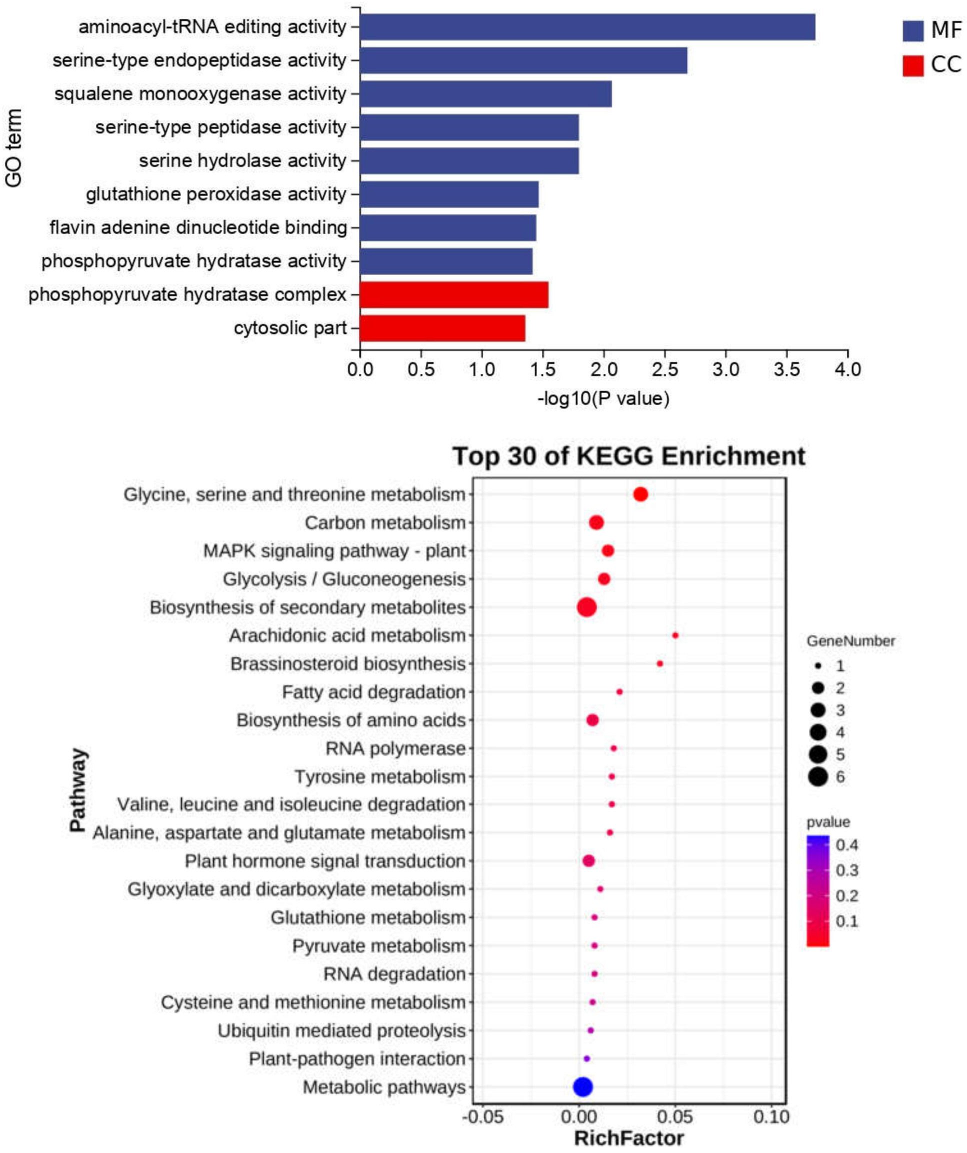


**Figure S6.** GO and KEGG enrichment analysis of protein coding genes of darkred module.
